# Association between serum 25‐hydroxyvitamin D concentrations and obesity in one‐year‐old Chinese infants

**DOI:** 10.1002/fsn3.2279

**Published:** 2021-05-03

**Authors:** Bingbing Guo, Yue Zhang, Jianan Lu, Shuang Guo, Yingtong Jiang, Jingjing Pei, Ran Wang, Chen Zhang, Haoyue Teng, Qiuyu Chen, Xinye Jiang, Jieyun Yin

**Affiliations:** ^1^ Department of Child Health Care The Affiliated Wuxi Maternity and Child Health Care Hospital of Nanjing Medical University Wuxi, Jiangsu China; ^2^ Department of Epidemiology and Biostatics Jiangsu Key Laboratory of Preventive and Translational Medicine for Geriatric Diseases School of Public Health Medical College of Soochow University Suzhou China

**Keywords:** BMI, BMI *Z*‐score, infant, obesity, vitamin D deficiency

## Abstract

Recent studies suggested that vitamin D is linked with obesity, but evidence in infants is scarce. Therefore, we aimed to make an exploration in infants. A total of 414 infants at one year old who visited Maternity and Child Health Care Hospital of Wuxi in China were recruited. Finger‐stick blood sampling was conducted in all the subjects, and serum 25‐hydroxyvitamin D [25(OH)D] concentrations were measured. Maternal characteristics during pregnancy and infantile information were collected by questionnaires or extracting from medical records. Multivariable linear models were performed to assess the relationship between 25(OH)D and body mass index (BMI), while multivariable logistic regression models were used to examine the association between 25(OH)D and obesity. Among the 414 infants, 69 (16.67%) and 81 (19.57%) infants were defined as obesity and vitamin D deficiency [25(OH)D < 50 nmol/L], respectively. The mean (*SD*) of 25(OH)D concentration was 68.05 (19.05) in infants without obesity, which was significantly higher than that of obese infants [60.36(18.49), *p* = .002]. Inverse linear relationships were observed between 25(OH)D level and BMI (*β* = −0.017, *p* = .004) as well as BMI *Z*‐score (*β* = −0.010, *p* = .004). Furthermore, vitamin D deficiency was associated with an increased risk of obesity of infants (adjusted odds ratio = 2.74, 95% confidence interval = 1.20–6.25, with 25(OH)D ≥ 75 nmol/L as a reference). The results showed that serum 25(OH)D concentrations were significantly lower in infants with obesity, suggesting vitamin D deficiency may be an independent risk factor for obesity among one‐year‐old Chinese infants.

## Graphical abstract

Serum 25‐hydroxyvitamin D concentrations were significantly lower in obese infants than their counterparts. Vitamin D levels < 50 nmol/L may be a risk factor for infantile obesity.

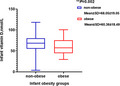

## 1 INTRODUCTION

Prevalence of overweight and obesity has been on the rise for decades and has become a globally serious public health issue (Jaacks, 2019). One combined meta‐analysis reported that the prevalence of overweight/obesity had an increase of 6.7% and 5.1% from 1991–1995 to 2011–2015, respectively. Besides, the rate of infancy obesity was up to 11.70% in China which ranked highest in the whole world (Guo, 2019). Obesity may affect the growth of children and contribute to poor cognitive function and altered timing of puberty (Wang, 2019). Without timely intervention, obese children are more likely to remain obese in adolescent and adulthood and are at elevated risks of metabolic disorders, type 2 diabetes, and cardiovascular diseases later in life (Hughes et al., 2014; Kim et al., 2017; Koyama, 2014; Rolland‐Cachera & Péneau, 2013). This underlines the importance of identification of modifiable, early risk factors of obesity.

In China, vitamin D deficiency is popular in the pediatric population (Li, 2020). Vitamin D plays a role in various physiologic and pathologic processes in the human body. It mainly affects bone health through regulating calcium and phosphorus absorption (Holick, 2002). Moreover, research has suggested that the metabolism, storage, and activation of vitamin D were influenced by adiposity (Shi et al., 2001). On the other hand, vitamin D functions as a preadipocyte inhibitor which may thus enhance adipogenesis and then contribute to the development of obesity (Shi et al., 2001). Therefore, some studies reported that vitamin D status is related with body mass index (BMI) in children and adolescents (Barja‐Fernández, 2018; Kumaratne et al., 2017; Li, 2020; Rodríguez‐Rodríguez et al., 2010; Turer et al., 2013), although controversial results also existed (Aypak et al., 2014; Creo et al., 2013; Hu, 2017; Mohammadian et al., 2014; Poomthavorn et al., 2012). Nevertheless, data are lacking regarding the association between vitamin D status and BMI in infants.

Thus, our present study aimed to evaluate vitamin D nutritional status and the association between serum 25‐hydroxyvitamin D [25(OH)D] and BMI in one‐year‐old Chinese infants.

## 2 METHODS

### 2.1 Subjects and study design

**FIGURE 1:**
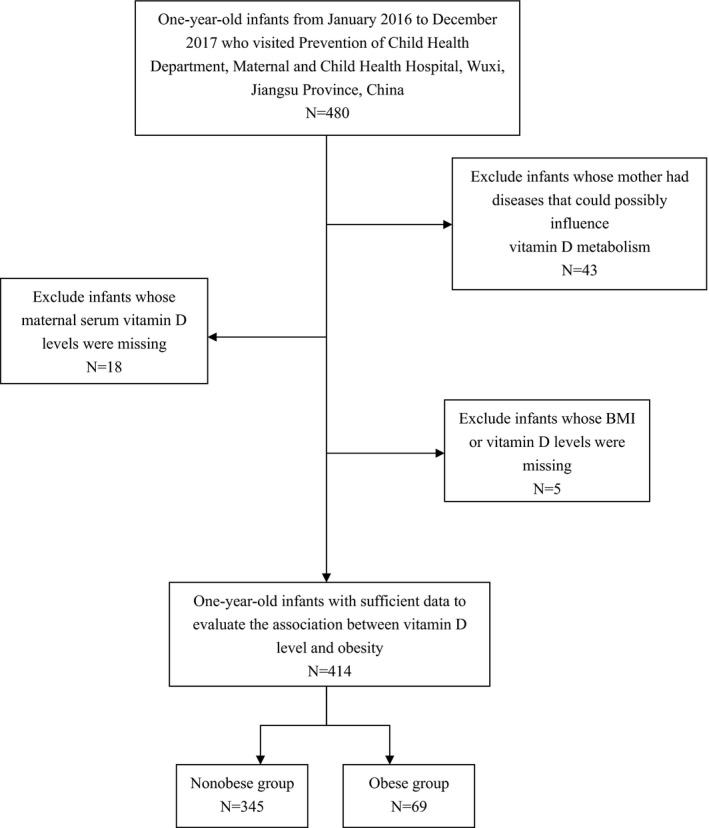
Flowchart for selection process of the study population

From January 2016 to December 2017, we recruited 480 one‐year‐old infants who consecutively visited Department of Child Health Prevention, the Affiliated Wuxi Maternity and Child Health Care Hospital of Nanjing Medical University, Jiangsu Province, China. Detailed information for infants and corresponding mothers was retrieved from medical records and through questionnaire interviews. Thereafter, 43 infants whose mother had diseases (i.e., liver and renal diseases) that could possibly influence vitamin D metabolism were excluded. Further exclusion was made for infants without BMI or vitamin D status (*n* = 5), as well as missing for maternal vitamin D status (*n* = 18). In final, 414 eligible infants were included. The flowchart of the selection process was illustrated in Figure 1.

### 2.2 Infant vitamin D examination

Detailed method was reported in previous study (Zhao, 2015). Briefly, 200μL finger‐sticking blood samples were collected from each participant and placed directly into a 0.5 milliliter micro‐tube. Within 10 min, the blood samples were centrifuged at 3,500 rpm for 15 min. Serum samples were stored at −80℃ until enzyme‐linked immunosorbent assay, the purpose of which was measuring for serum 25(OH)D levels (IDS Ltd.). The interassay and intra‐assay coefficients of variation were <10%, respectively.

Although the optimal vitamin D level is surrounded by debate, vitamin D deficiency has been historically defined and recommended by Endocrine Society's clinical practice guidelines as <50 nmol/L (Holick, 2006, 2007; Holick et al., 2011). Meanwhile, to maximize the effect of vitamin D on bone and extra‐skeletal health, it was suggested that the level of 25(OH)D should be above 75 nmol/L. (Holick et al., 2011). Additionally, 25(OH)D < 50, 50–75, and ≥75 nmol/L as deficiency, insufficiency, and sufficiency were often used in Chinese population (Guo, 2018; Zhang et al., 2013; Zhu, 2012). Thus, vitamin D nutritional status was assessed by 25(OH)D concentration as “deficiency” (<50 nmol/L), “inadequacy” (50 to <75 nmol/L), and “sufficiency” (≥75 nmol/L) in the current study, respectively.

The seasons of specimens collection were divided into spring (from March to May), summer (from June to August), autumn (from September to November), and winter (from December to February).

### 2.3 Infant anthropometric data

Recruited infants underwent a clinical examination for their essential information such as current height and weight. Duplicate measurements were made, and averaged values were recorded. Weight and length were measured with infants in light clothing and without shoes. Weight was measured using a digital scale and length was measured from the top of head to feet using an infant mat. Infants whose BMI ≥95th percentiles for each sex were considered to be obese according to the World Health Organization (WHO) growth standards (BMI ≥19.7 as obesity for one‐year‐old boys and BMI ≥19.6 as obesity for one‐year‐old girls; WHO, 2014). BMI *Z*‐score expresses the number of *SD*s below or above the reference mean value for BMI. The formula for calculating the BMI *Z*‐score was (*X* − m)/*SD*, in which *X* was the observed value of BMI, *m* and *SD* referred to the mean and standard deviation value of the distribution corresponding to the reference population by WHO standards for child growth (WHO, 2014). Macros in SAS were also provided by WHO and used to calculate BMI *Z*‐score in our study (https://www.who.int/tools/child‐growth‐standards/software).

### 2.4 Details of infant characteristics

Neonatal characteristics were retrieved from medical records, including delivery mode, gestational week, weight, and length at birth.

Infantile characteristics after birth were also collected through questionnaire interview, such as breastfeeding duration, outdoor time per day, and vitamin D supplementation. Breastfeeding duration was divided into four categories (≥6, 3–6, 0‐3 months and none). Besides, outdoor time per day were classified into none, 0–1, and ≥1 hr per day.

### 2.5 Details of maternal characteristics

Maternal characteristics were extracted from medical records or through questionnaire interview, such as maternal age at delivery, pre‐pregnancy BMI, educational level, family income level, passive smoking, vitamin D, and folic acid supplementation during pregnancy, serum vitamin D [25(OH)D] levels between 17th and 29th weeks of gestation, gestational weight gain (GWG) during pregnancy, and gestational complications (i.e., gestational diabetes, hypertensive disorders during pregnancy and hyperlipidemia). GWG was measured as the difference of weight before pregnancy and at delivery.

Pregnancy BMI level was divided into lean (<18.5 kg/m^2^), normal (18.5 to <24 kg/m^2^), overweight (24 to <28 kg/m^2^), and obese (≥28 kg/m^2^; Zhou & Cooperative Meta‐Analysis Group of the Working Group on Obesity in China, 2002). GWG status was stratified according to Institution of Medicine (IOM) GWG guidelines as inadequate, adequate, and excessive (Institute of Medicine (US) et al., 2009).

### 2.6 Missing data

Inevitably, there remained missing values for maternal and infantile characteristics. The percentage of missing covariates varied from 0.24% to 1.9%. In spite of the existence of these missing covariates, the main observed factors including maternal and infantile vitamin D and obesity status were complete. Also, considering the random type and small percentage of our missing data, they were handled by complete case analysis which was simpler to operate and not necessarily lead to biased results (Mukaka, 2016; Pigott, 2001). A summary of number/percentage of missing values in the logistic model was described in Table S1.

### 2.7 Statistical analysis

Continuous and categorical variables were presented as mean ± *SD* and frequency (percentage), respectively. The difference of characteristics between obese and nonobese infants was compared using Student's *t* test for continuous variables and chi‐square test for categorical variables, respectively. Linear regression models were used to examine the relationship between 25(OH)D and one‐year‐old infant BMI as well as BMI Z‐score. Model 1 was unadjusted. Model 2 was adjusted for potential confounding factors which could influence infantile vitamin D levels including sex, season of infant serum sampling, infant outdoor time, maternal vitamin D level, maternal vitamin D, and folic acid supplementation. Model 3 additionally controlled for obesity‐related factors comprised of pre‐pregnancy BMI categories, GWG status, maternal age, birth weight, breasting feeding duration, and passive smoking. Logistic regression models were also performed to evaluate the association between infant vitamin D nutritional status and obesity, with the same covariates in the linear regression models. Likelihood ratio tests were used to assess linear trends in ORs over the vitamin D groups, scoring sufficiency, inadequacy, and deficiency as 1, 2, and 3.

All *p* values were two‐tailed, and *p* <.05 was defined as statistically significant. SAS (SAS Institute Inc.) was used for all statistical analyses.

## 3 RESULTS

### 3.1 Baseline characteristics

The current study enrolled 69 obese and 345 nonobese infants at one year old. It was found that 140 (33.81%), 193 (46.62%), and 81 (19.57%) infants had sufficient, inadequate, and deficient vitamin D, respectively. Table 1 shows the characteristics of infant‐mother pairs according to infantile obese status. The mean maternal age at delivery was 30.96 ± 4.93 years old in the obese group, older than its counterparts (29.30 ± 4.24, *p* =.012). Maternal 25(OH)D levels of obese infants were significantly lower than that in nonobese infants (*p* =.043). Mothers with higher preconceptional BMI (*p* =.029), had no supplementation for vitamin D (*p* =.011) or folic acid (*p* =.001), and exposed to passive smoking (*p* <.001) during pregnancy tented to have obese babies. Also, infants who were boys (*p* <.001), had shorter breastfeeding duration (*p* =.030), or spent less time outdoors (*p* =.015) were more likely to suffer obesity.

**TABLE 1:** Characteristics of infants and corresponding mothers

Variable	Total (*N* = 414)	Nonobese (*n* = 345)	Obese (*n* = 69)	*p* value
***Maternal characteristics***				
Maternal age at delivery, years	29.58 ± 4.40	29.30 ± 4.24	30.96 ± 4.93	0.012*
Delivery, *n* (%)				0.371
Vaginal	183 (44.63)	156 (45.61)	27 (39.71)	
Cesarean	227 (55.37)	186 (54.39)	41 (60.29)	
25(OH)D, nmol/L	35.86 ± 15.41	36.49 ± 15.85	32.46 ± 12.35	0.043^*^
Vitamin D supplementation, *n* (%)	104 (25.12)	95 (27.54)	9 (13.04)	0.011^*^
Folic acid supplementation, *n* (%)	264 (63.77)	232 (67.25)	32 (46.38)	0.001^**^
Passive smoking status, *n* (%)				<0.001^***^
Yes	290 (70.05)	256 (74.20)	34 (49.28)	
No	124 (29.95)	89 (25.80)	35 (50.72)	
Gestational weight gain status, *n* (%)				0.144
Inadequate	112 (27.52)	97 (28.61)	15 (22.06)	
Adequate	184 (45.21)	156 (46.02)	28 (41.18)	
Excessive	111 (27.27)	86 (25.37)	25 (36.76)	
Pre‐pregnancy BMI categories, *n* (%)				0.029^**^
Lean (<18.5 kg/m^2^)	69 (16.71)	61 (17.68)	8 (11.76)	
Normal (<24 kg/m^2^)	281 (68.04)	239 (69.28)	42 (61.76)	
Overweight (24 to <28 kg/m^2^)	49 (11.86)	34 (9.86)	15 (22.06)	
Obese (≥28 kg/m^2^)	14 (3.39)	11 (3.19)	3 (4.41)	
Gestational diabetes, *n* (%)	97 (23.43)	78 (22.61)	19 (27.54)	0.378
Hypertensive disorders during pregnancy, *n* (%)	16 (3.86)	13 (3.77)	3 (4.35)	0.820
Gestational hyperlipidemia, *n* (%)	3 (0.72)	3 (0.87)	0 (0.00)	0.437
Education level, *n* (%)				0.091
Primary school or below	5 (1.22)	5 (1.46)	0 (0.00)	
Junior school	30 (7.32)	29 (8.48)	1 (1.47)	
High school	55 (13.41)	48 (14.04)	7 (10.29)	
University and above	320 (78.05)	259 (76.02)	61 (88.24)	
Family income level, *n* (%)				0.395
Poor	15 (3.96)	14 (4.42)	1 (1.61)	
Normal	196 (51.72)	168 (53.00)	28 (45.16)	
Good	147 (38.79)	118 (37.22)	29 (46.78)	
Very good	21 (5.54)	17 (5.36)	4 (6.45)	
***Child characteristics***				
Sex, *n* (%)				<0.001^***^
Male	215 (51.93)	164 (47.54)	51 (73.91)	
Female	199 (48.07)	181 (52.46)	18 (26.09)	
25(OH)D, *n* (%)				<0.001^***^
Deficiency (<50 nmol/L)	81 (19.57)	55 (15.94)	26 (37.68)	
Inadequacy (50 to <75 nmol/L)	193 (46.62)	168 (48.70)	25 (36.23)	
Sufficiency (≥75 nmol/L)	140 (33.81)	122 (35.36)	18 (26.09)	
Birth weight, kg	3.39 ± 2.43	3.26 ± 0.84	4.03 ± 5.65	0.265
Gestational age, weeks	38.33 ± 2.03	38.39 ± 1.92	37.95 ± 2.59	0.247
Season of serum sampling, *n* (%)				0.535
Spring	75 (18.12)	65 (18.84)	10 (14.49)	
Summer	122 (29.47)	97 (28.12)	25 (36.23)	
Autumn	70 (16.91)	60 (17.39)	10 (14.49)	
Winter	147 (35.51)	123 (35.65)	24 (34.78)	
Breastfeeding duration (months), *n* (%)				0.030^*^
≥6	173 (43.25)	155 (46.55)	18 (26.87)	
3–6	45 (11.25)	36 (10.81)	9 (13.43)	
0‐3	33 (8.25)	26 (7.81)	7 (10.45)	
0	149 (37.25)	116 (34.83)	33 (49.25)	
Outdoor time (h/day), *n* (%)				<0.001^***^
None	77 (18.87)	51 (15.04)	26 (37.68)	
0–1	220 (53.92)	190 (56.05)	30 (43.48)	
≥1	111 (27.21)	98 (28.91)	13 (18.84)	
Vitamin D supplementation, *n* (%)	400 (98.04)	333 (97.04)	67 (98.53)	0.201

Statistically significant values are indicated in bold as followings: ^*^ *p* < 0.05, ^**^ *p* < 0.01, ^***^ *p* < 0.001.

### 3.2 Linear relationship between 25(OH)D and BMI as well as BMI *Z*‐score

**FIGURE 2:**
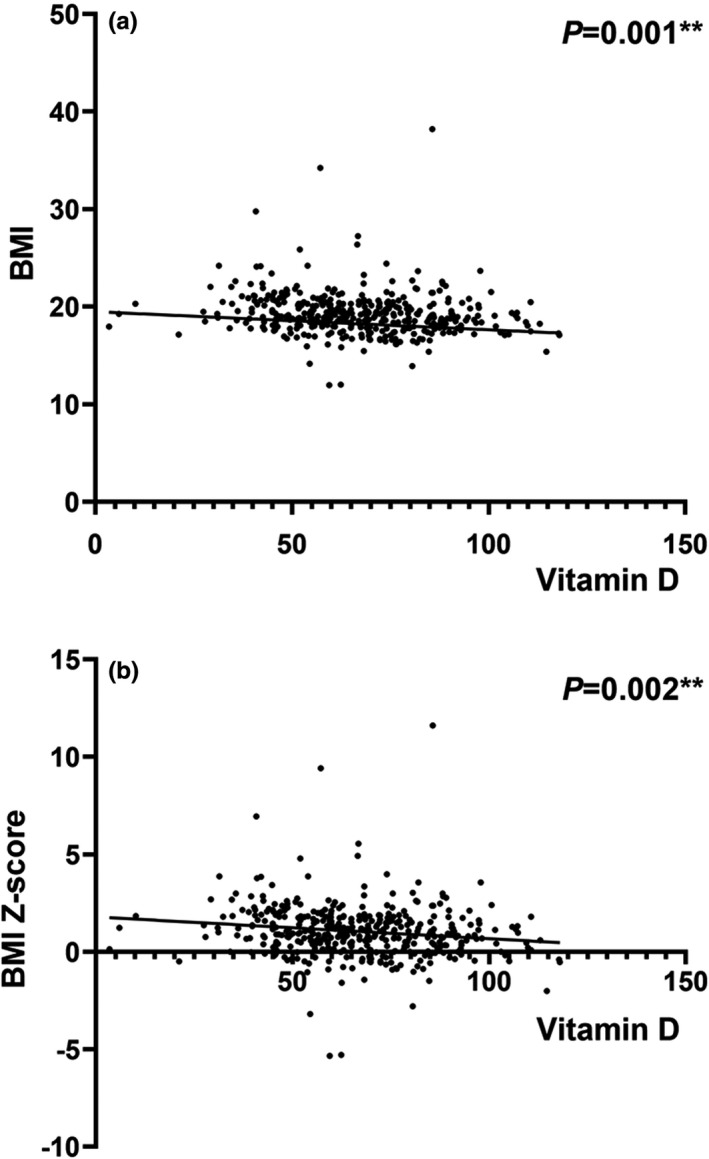
Scatter plot of the linear relationship between vitamin D (nmol/L) and BMI (a) as well as BMI *Z*‐score (b). BMI, body mass index.

Table 2 presents the linear relationship between 25(OH)D concentrations and BMI as well as BMI *Z*‐score in one‐year‐old infants. In Model 1, an inverse relation between 25(OH)D and BMI was found (*β* = −0.019, *p* =.001). Figure 2 gives an overview of the trend in the linear association between vitamin D and BMI as well as BMI *Z*‐score. The correlation remained significant after adjustment for covariates in Model 2 (*β* = −0.018, *p* =.002) and Model 3 (*β* = −0.017, *p* =.004). When it turned to BMI Z‐score, similar results were found.

**TABLE 2:** Linear relationship between 25(OH)D levels and BMI or BMI Z‐score

Dependent variables	Model 1		Model 2		Model 3	
	BMI	BMI *Z*‐score	BMI	BMI *Z*‐score	BMI	BMI *Z*‐score
*β* estimate (SE)	−0.019 (0.006)	−0.011 (0.004)	−0.018 (0.006)	−0.011 (0.003)	−0.017 (0.006)	−0.010(0.004)
*p* value	0.001^**^	0.002^**^	0.002^**^	0.002^**^	0.004^**^	0.004^**^

Note

Model 1 was unadjusted;

Model 2: adjusted for sex, season of infant serum sampling, infant outdoor time, maternal 25(OH)D level, maternal vitamin D, and folic acid supplementation;

Model 3: based on model 2, additionally adjusted for pre‐pregnancy BMI categories, GWG status, maternal age, birth weight, breasting feeding duration, and passive smoking.

Abbreviations: SE, standard error; BMI, body mass index; GWG, gestational weight gain.

statistically significant values are indicated in bold as followings: ^*^ *p* < 0.05, ^**^ *p* < 0.01, ^***^ *p* < 0.001.

### 3.3 Association between 25(OH)D concentrations and infant obesity status

**FIGURE 3:**
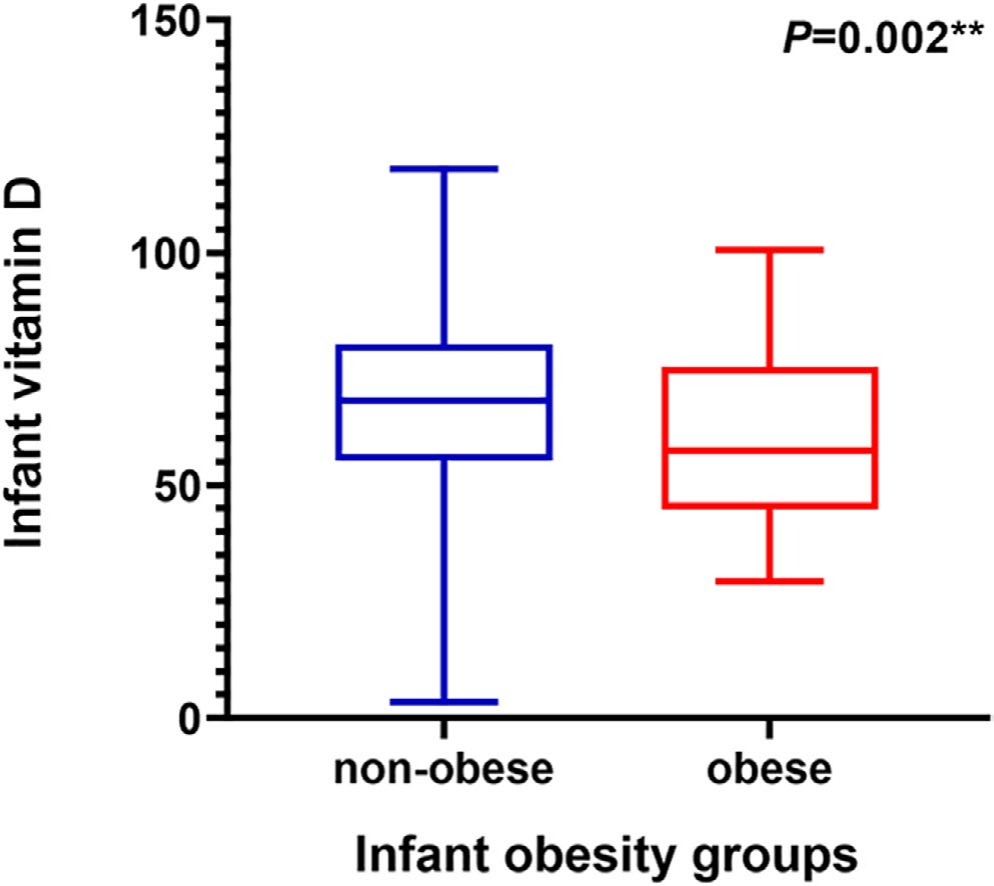
Box plot of infant serum 25(OH)D concentrations (nmol/L) according to infantile obese status

As illustrated in Figure 3, the mean (*SD*) of 25(OH)D concentration was 68.05(19.05) nmol/L in nonobese infants, while that for the obese group was significantly lower [mean (*SD*) = 60.36 (18.49), *p* =.002].

The probability of becoming obese was revealed by logistic regression models using vitamin D sufficiency as a reference (Table 3). It was found that infant vitamin D deficiency was associated with increased risk of obesity in the crude model, with OR (95% CI) equaled 3.20 (1.62–6.33). However, the association between vitamin D inadequacy and obesity did not reach statistical significance, with OR (95% CI) of 1.01 (0.53–1.93). After adjusting for comprehensive covariates in Model 3, significant association between infant vitamin D deficiency and obesity remained (OR = 2.74, 95% CI = 1.20–6.25). However, no statistically significant association was yielded for vitamin D inadequacy (OR = 0.89, 95% CI = 0.42–1.90).

**TABLE 3:** Association between 25(OH)D groups and obesity status in one‐year‐old infants

Groups	Obese, *n* (%)	Nonobese, *n* (%)	Model 1		Model 2		Model 3	
			OR (95% CI)	*p* value	OR (95% CI)	*p* value	OR (95% CI)	*p* value
Sufficiency	122 (35.36)	18 (26.09)	1.00		1.00		1.00	
Inadequacy	168 (48.70)	25 (36.23)	1.01 (0.53–1.93)	0.979	0.93 (0.46–1.88)	0.833	0.89 (0.42–1.90)	0.761
Deficiency	55 (15.94)	26 (37.68)	3.20 (1.62–6.33)	0.001^**^	3.12 (1.48–6.61)	0.003^**^	2.74 (1.20–6.25)	0.017^*^
*p*_for trend_				<0.001^***^		0.001^**^		0.009^**^

Note

Model 1 was unadjusted;

Model 2: adjusted for sex, season of infant serum sampling, infant outdoor time, maternal vitamin D level, maternal vitamin D, and folic acid supplementation;

Model 3: based on model 2, additionally adjusted for pre‐pregnancy BMI categories, GWG status, maternal age, birth weight, breasting feeding duration, and passive smoking.

Abbreviations: BMI, body mass index; GWG, gestational weight gain.

Statistically significant values are indicated in bold as followings: ^*^ *p* < 0.05, ^**^ *p* < 0.01, ^***^ *p* < 0.001.

## 4 DISCUSSION

In our study of Chinese one‐year‐old infants, the prevalence of vitamin D deficiency was 19.57%. Slightly inverse linear relationships were found between 25(OH)D and BMI as well as BMI *Z*‐score, even after adjusting for comprehensive maternal and infantile variables. Besides, vitamin D deficiency, but not vitamin D inadequacy, was independently associated with increased risk of obesity.

Our study elucidated that elder maternal age, higher pre‐pregnancy BMI, shorter breastfeeding duration, and limited outdoor time were tied with higher probability of infant obesity. In line with our results, previous studies have reported that risk of obesity was high in children whose mothers had preconceptional overweight or obese (Cebeci & Guven, 2015; Edlow, 2017; Sanchez, 2018). Research also suggested that breastfeeding may be a protective factor for childhood obesity (Marseglia, 2015). Besides, our findings showed that limited outdoor time tended to be a risk factor for infant obesity. Outdoor experiences are critical for infant to develop fit bodies (McCurdy et al., 2010). Vitamin D was mainly produced by skin exposure to solar ultraviolet B radiation, which is related to outdoor behavior. Limited outdoor time may decrease endogenous synthesis of vitamin D (Verbraecken et al., 2006) and thus related with high risk of obesity. Our findings supported the view that adequate sunshine exposure and vitamin D supplementation should be encouraged in infants.

A variety of studies have looked into the role of vitamin D acting in obesity. Adipocyte was found to be the storage organ for vitamin D (Wortsman et al., 2000). In adipocytes, vitamin D exerts an inhibition effect on adipogenesis by combining to vitamin D receptor on the membrane (mVDR; Abbas, 2017). The combination of vitamin D and mVDRs could particularly affect differentiation of preadipocytes, thus exerts an anti‐obesity role (Narvaez et al., 2009). Hence, lower 25(OH)D concentrations may inversely upregulate preadipocyte differentiation and contribute to future obesity. Additionally, in obese subjects, higher content of body fat could provide more storage space for vitamin D and in turn lower vitamin D bioavailability in circulation (Zhao, 2015). However, there remained some disagreements. A mendelian randomization study reported a causal relationship that a higher BMI may lead to a decreased level of vitamin D, while the effect of vitamin D on BMI was small (Vimaleswaran, 2013). It should be noted that there existed several differences in the characteristics of the population between their study and ours. Subjects enrolled in their study were mainly adults and came from various countries, while participants in ours were Chinese infants located in areas with sufficient sunlight. Differences in age, ethnicity, geographical environments, and year of birth of the population may contribute to the discrepancy. What's more, a recent meta‐analysis of several randomized controlled trials suggested that vitamin D supplementation was related to greater weight loss, which indicated an inverse relationship between vitamin D and obesity (Perna, 2019). Mendelian randomization study, however, was a statistical method that had a relatively lower capacity to verify the causal relationship. Hence, further studies should be conducted to confirm the causal relationship between vitamin D and obesity.

Notably, our study found no association between vitamin D inadequacy and infant obesity. Despite the mechanism was unclear, we could speculate that it may be due to the compensation for decreasing vitamin D status in the early stage. This offers us a clue that timely intervention such as supplementation for vitamin D once the decline is spotted may bring benefits for childhood obesity prevention. However, there is no consensus on the exact dosage of vitamin D intakes on protecting against childhood obesity. Specifically, a recent meta‐analysis suggested that the favorable effect of vitamin D supplementation on cardiometabolic outcomes in children may only be shown when serum 25(OH)D levels achieved >70 nmol/L or an increase of 20 nmol/L (Hauger, 2020).

There remained several limitations that should be admitted in our study. Firstly, the sample size of our study was relatively small. However, with a sample size of 414, the current study had a statistical power of 90.2% to measure the difference of 25(OH)D concentrations between obese and nonobese infants. Secondly, infants included in our study were mainly from low altitude southeastern areas of China, whose sun exposure was abundant all year round. The limited geographic environments made it difficult to reflect infant vitamin D status in other parts of China. Thirdly, some potential confounding factors that could possibly influence the association between vitamin D and obesity may not be assessed in the current study. Last, due to the nature of observational study, we cannot infer the causal relationship between vitamin D and obesity. Further studies are warrant for validation.

## 5 CONCLUSION

Overall, the current study based on a Chinese infant population suggested that 25(OH)D levels <50 nmol/L were independently associated with higher risk of infant obesity.

## CONFLICT OF INTEREST

The authors declare that they do not have any conflict of interest.

## ETHICAL REVIEW

This study conformed to the Declaration of Helsinki, US, and European Medicines Agency Guidelines for human subjects. This study was approved by the ethics committee of the Affiliated Wuxi Maternity and Child Health Care Hospital of Nanjing Medical University. The date and number of the approval were 2020.03.12 and 202106031208, respectively.

## INFORMED CONSENT

Written informed consent was obtained from all study participants.

## Supporting information

Table S1

## Data Availability

All data used in the study are available in the submitted article.
