# Supplementary material for: Association between serum 25‐hydroxyvitamin D concentrations and obesity in one‐year‐old Chinese infants
Source: Food Sci Nutr. 2021 May 3;9(6):3191–9. doi: 10.1002/fsn3.2279 (PMC8194735; doi:10.1002/fsn3.2279)
Supplement: Supplementary file 1 — Table S1 [file FSN3-9-3191-s001.docx]

Supplementary Table 1. The number (percentage) of missing data for confounding covariates

| Covariates | Missing data (%) |
| --- | --- |
| **Maternal characteristics**  Maternal age at delivery | 8(1.9) |
| Delivery | 4(0.97) |
| 25(OH)D | 0(0) |
| Vitamin D supplementation | 0(0) |
| Folic acid supplementation | 0(0) |
| Passive smoking | 0(0) |
| Gestational weight gain status | 7(1.70) |
| Pre-pregnancy BMI categories | 1(0.24) |
| Gestational diabetes | 0(0) |
| Hypertensive disorders during pregnancy | 0(0) |
| Gestational hyperlipidemia | 0(0) |
| Education level | 4(0.97) |
| **Child characteristics** |  |
| Sex | 0(0) |
| 25(OH)D | 0(0) |
| Birth weight | 0(0) |
| Season of serum sampling | 0(0) |
| Breasting feeding duration | 14(3.4) |
| Outdoor time | 6(1.45) |
| Vitamin D supplementation | 0(0) |
